# Current Advances on the Important Roles of Enhancer RNAs in Gene Regulation and Cancer

**DOI:** 10.1155/2018/2405351

**Published:** 2018-05-22

**Authors:** Yuhan Liu, Mengting Ding, Qunjun Gao, Anbang He, Yuchen Liu, Hongbing Mei

**Affiliations:** ^1^Department of Urology, Shenzhen Second People's Hospital, The First Affiliated Hospital of Shenzhen University, Shenzhen 518000, China; ^2^Department of Urology, Shenzhen Second People's Hospital, Clinical Medicine College of Anhui Medical University, Shenzhen 518000, Guangdong, China; ^3^Department of Urology, Shenzhen Second People's Hospital, Graduate School of Guangzhou Medical University, Guangzhou Medical University, Guangzhou 511436, China; ^4^Department of Urology, Peking University First Hospital, The Institute of Urology, Peking University, National Urological Cancer Centre, Beijing 100034, China

## Abstract

Revealing the gene regulation networks governing cancer initiation and development is necessary while it remains uncompleted. In recent years, enhancers have been reported to be widely transcribed, resulting in the generation of enhancer RNAs (eRNAs). Previous studies have reported that eRNAs are a subclass of long noncoding RNAs (lncRNAs), which play a critical role in gene regulation and cancer development. These eRNAs can promote enhancer-promoter (E-P) looping formation by binding to other protein factors or propel expression of downstream protein-coding gene. In this review, we have focused on the characteristics of eRNAs and illustrated the biological function and potential mechanism of eRNAs in regulating gene expression and cancer development.

## 1. Introduction

It is generally thought that regulation of gene expression is required for all sorts of biological processes, including cell proliferation, differentiation, and disease progression [1–5]. Particularly, regulatory DNA regions are often identified to act on transcription units, just like the widespread transcription happening on enhancers [6–10]. Multiple studies of the enhancer-derived RNAs and their contribution to gene regulatory processes may greatly enrich our knowledge of the composition and functional operation of the genome [11–13].

Enhancer was initially described as distal cis-regulatory DNA elements of gene activation in the simian virus 40 (SV40) genome in 1980s [14]. Enhancers are 72 bp-long SV40 DNA fragment, which can significantly activate transcription from the promoter of its target gene [14–16]. Enhancers have an effect on long distances of thousands or hundreds of thousands of base pairs, including upstream, downstream, and a transcription unit [17–24]. Recent studies reported that the human genome harbors millions of enhancers that can be activated at different developmental stages and in various tissues and cell types [25–27].

In 2010, high-throughput sequencing was adapted to characterize transcriptional enhancers by Kim et al. [28], which revealed bidirectional RNA transcription originating from thousands of chromatin state-defined enhancers. Enhancers with stronger activity were characterized by additional abundance of RNA polymerase II (RNA Pol II) and production of enhancer RNAs (eRNAs) [28, 29]. They identified these RNAs as enhancer RNAs (eRNAs). eRNAs are limited to around 500–2000 bp and arise from bidirectional transcription of both strands of DNA from a central nontranscribed region. Intriguingly, emerging studies revealed a close association between enhancer activation and eRNAs transcription and demonstrated that eRNAs can promote E-P looping formation by binding other protein factors, as well as propelling the expression of downstream gene [28, 30–33]. Since enhancers are important in regulating gene expression from target promoters and defined the potential hierarchical relationship between promoters, which means that eRNAs serve as an critical transcriptional regulator in polygenic complexes, these studies can help us to better understand regulatory network, and aberrant eRNAs regulations have been reported to be related to tumorigenesis. Herein, we describe recent advances in our understanding of eRNAs function in gene regulation and development of cancer.

## 2. Enhancer RNAs and Their Characteristics

Enhancer RNAs are clearly distinguishable from the long noncoding RNAs (lncRNAs) and microRNAs (miRNAs) whose functions have been better characterized. Firstly, these eRNAs are limited to around 500–2000 bp and arise from bidirectional transcription of both strands of DNA from a central nontranscribed region [23, 28, 34, 35]. Secondly, they mostly have nonpolyadenylated and unspliced tails, suggesting that they have very short half-lives, when compared with other transcription units in the genome. The abundance of eRNAs is 19–34-fold lower than mRNAs of neighboring protein-coding genes [36]. Thirdly, most mRNAs are stable, multiexonic, long transcripts and exported to the cytoplasm. In contrast, eRNAs are sensitive to exosome-mediated decay, relatively shorter, and retained in the nucleus [36, 37]. Fourthly, eRNAs have been found to transcribe from hypothetical enhancer regions distinguished by high levels of H3K4me1 and H3K4me2 compared with the H3K4me3 [38]. Furthermore, eRNAs often accumulate near the start site of transcription of target genes, where they can stabilize E-P interactions [39, 40] and transcription factor (TF) binding [41].

## 3. Enhancer RNAs in Gene Regulation

**Figure 1:**
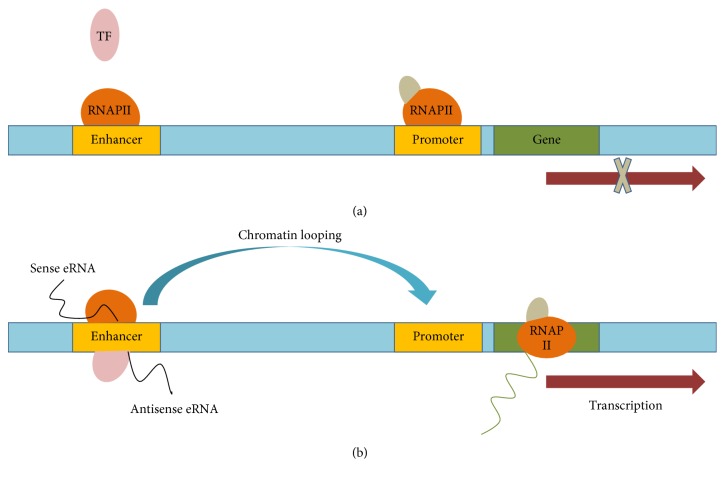
Schematic representation of eRNA activation and function. Transcriptional activation of enhancer domains is typically triggered by binding to transcription factor (TF). Enhancer RNAs could functionally contribute to gene activation by modulating the stability of enhancer : promoter (E : P) looping via interacting with looping factors.

Without a doubt, the functions of eRNAs afford novel visual angle to the processes of spatiotemporal gene regulation, chromosomal interactions, and enhancer activity (Figure 1). It has been suggested that eRNAs may function as transcription activators by cloning different enhancer genomic fragments [34, 35, 42–44]. Lam et al. [41] cloned different sizes of genomic fragments from an endogenous enhancer locus and demonstrated that the enhancer construct containing eRNA-coding sequences had sufficient enhancer activity and higher transcriptional activity, especially the core enhancer fragments containing lineage-determining transcription factors (LDTF) binding sites. Moreover, the extra effect from the eRNA would be shut down while the direction of its coding sequence was inversed compared to the central enhancer. Contrary to the persistence of putative TF binding sites, the sequence of the eRNA product completely changes due to this inversion, which indicates that the sequence of the eRNA is critical for its function. eRNA was also seen to play important role in the maintenance of enhancer-promoter contacts. The prevailing idea have put forward that eRNAs may affect the accessibility of chromatin and RNAPII association with the core promoter elements of the protein-coding gene [11, 45]; more importantly, these experiments have revealed an effect specifically on histone modifications [12, 46]. However, the histone modification would unlikely be involved with the whole genes due to the fact that it can be influenced by different mechanisms and pathways in genomic transcription.

Several eRNAs appear to exert their effects on transcriptional elongation. The induction of neuronal immediate early genes was found to rely on the ability of eRNAs to induce release of the negative elongation factor (NELF) from paused RNAPII at their target gene promoters, without affecting RNAPII recruitment or DNA looping [30]. This interruption by RNAPII is deemed to allow rapid and synchronous gene expression, while first enabling the establishment of a suitable chromatin environment for transcription to proceed. RNAP interruption is common in many mammalian genes, particularly at genes induced by specific signals [31], implicating that this function of the eRNA is likely highly context-specific. Furthermore, some eRNAs were thought to help maintain certain RNA or DNA binding transcription factors at regulatory elements [32].

## 4. Enhancer RNAs and Tumorigenesis

Enhancer RNAs may also act as epigenetic regulators that maintain an active chromatin state at transcribed gene loci. A better understanding of the molecular mechanisms of eRNAs by which they function in the normal and tumor cell will make better understanding of tumor pathogenesis. Alteration of eRNAs were correlated with tumorigenesis [47, 48]. Recent studies of aberrant eRNAs in various cancer types and their fundamental characteristics have been presented in Table 1.

## 5. Enhancer RNAs and Breast Cancer

Functional eRNAs were also found with the estrogen receptor *α* (ER-*α*) in human breast cancer cells [47]. By treating human breast cancer cells with 17 *β*-estradiol (E2), Li et al. observed a strong correlation between ER-*α*-bound enhancers, eRNA production, and the ER dependent regulation of neighboring protein-coding genes. Moreover, they identified that FOXC1, TFF1, or CA12, as E2-associated enhancers regulated eRNAs, which significantly repressed transcription of the near protein-coding genes. Together, they reported that knockdown of eRNAs adjacent to the PGR, NRIP1, SMAD7, KCNK5, P2RY2, SIAH2, and GREB1 genes may decrease the expression of eRNA transcript posttranscriptionally, but not the level of nascent transcription. Specially, loss-of-function of NRIP1e eRNA led to a significant reduction in the interactions between the* NRIP1 and TFF1 *loci, suggesting eRNA potential role in E2-mediated colocalization. Therefore, they concluded that induced eRNA production are involved in the actions of estrogen-associated gene enhancers, which contribute to E2-regulated gene activation by maintaining E2/ER*α*/eRNA-induced E-P looping.

Another studies demonstrated that nutlin-3a (an activator of p53) could induce eRNAs transcription in human breast cancer [49], just like estrogen. In MCF-7 cells, Léveillé et al. reported that lncRNA activator of enhancer domains (LED) was located at an enhancer region within CDKN1A gene, a potent p53-responsive cell cycle inhibitor, could bind to p53-regulated enhancer regions, which activated strong enhancers, and induced transcription of eRNAs, including P21, TOB1, PRKAG2, and SUFU. LED inhibition represses CDKN1A enhancer induction and activity and cell cycle arrest following p53 activation. These results uncovered a potential tumor inhibitor mechanism with a p53-regulated eRNA acting on enhancers and an burgeoning regulatory network with potential effect on tumor progression.

## 6. Enhancer RNAs and Castration-Resistant Prostate Cancer

In castration-resistant prostate cancer (CRPC) cells, bidirectional eRNAs production is induced after binding of androgen receptor (AR) to responsive enhancers and is concomitant with sustaining an open chromatin structure and indirect activation of gene expression possibly via the mechanism described above [50]. van der Steen et al. [51] reported that upregulated expression of the androgen receptor is pivotal for CRPC progression. Zhao et al. [52] found a group of AR-regulated enhancer RNAs that are upregulated in CRPC cells and patient tissues. They found PSA eRNA can* trans-*regulate expression of a subclass of genes involved in androgen action and cancer progression. PSA eRNA could combine with CYCLIN T1, stimulate P-TEFb, and boost* cis-* and* trans-*target gene transcription by raising Pol II-Ser2p (serine-2 phosphorylation of RNA polymerase II). They identified an HIV-1 TAR-L (TAR RNA-like) motif in PSA eRNA, which is necessary for P-TEFb activation and CRPC growth. While AR signaling strongly* trans*-activated one of the genes, the PSA gene generates eRNAs from its enhancer to amplify AR action by* trans*-regulating its downstream genes. In their research, they have explored a P-TEFb activation mechanism and showed that AR function was significantly associated with aberrant eRNAs and may be a potential biomarker in CRPC.

Another research has identified altered expression of AR-eRNAs in CRPC cells sensitive or resistant to enzalutamide (a second-generation androgen inhibitor) [53] and revealed that expression of AR-eRNAs and related mRNAs in the loci of* FTO, LUZP2, MARC1, and NCAM2* may be associated with enzalutamide resistance. They showed that eRNA expression was increased after long-term treated with enzalutamide, which is similar to the role of enzalutamide treatment in the interaction between its enhancer and promoter in CRPC cells. These results indicated that eRNAs alteration profiling is a viable approach to understand the role of eRNAs in enzalutamide resistance and to identify novel therapeutic targets for CRPC therapy.

## 7. Enhancer RNAs and Colorectal Cancer

McCleland et al. [54] developed a CRISPR loss-of-function screen and conducted a high-throughput screen to recognize an epigenetic modulators including bromodomain-containing protein 4 (BRD4), which revealed that BRD4 is involving in colon cancer cell proliferation, and its knockdown resulted in differentiation and growth arrest in the epigenetically dysregulated CpG island methylator phenotype (CIMP^+^) class of cancers. They identified a definite superenhancer in CIMP^+^ colon cancers that regulates cMYC transcription. They also reported that lncRNA CCAT1 (long noncoding RNA colon cancer-associated transcript 1) is transcribed from this superenhancer and is significantly sensitive to bromodomain and extraterminal (BET) inhibition. To directly investigate BRD4-specific effects as compared with BET-driven changes, they used whole-genome transcriptional profiling in HT-29 and HCT 116 cells after BRD4 knockdown or JQ1 (a BET inhibitor) inhibition, suggesting that CCAT1 is an immediate BET transcriptional target. Consistent with this superenhancer driven cMYC transcription, JQ1 treatment preferentially reduced cMYC expression in CCAT1-expressing cells. The CCAT1 eRNA itself has been previously shown to regulate cMYC expression, albeit at lower levels than those seen with BET inhibitors [55, 56]. Taken together, these results suggest that both BET activity and eRNA expression are necessary to drive cMYC transcription in cancers expressing CCAT1 and propose that CCAT1 is a novel diagnostic biomarker for identifying patients who are likely to benefit from BET inhibitors.

## 8. Enhancer RNAs and Basal Cell Carcinoma

Bal et al. [57] performed high-throughput sequencing to define mutations in the ACTRT1 gene, which encodes actin-related protein T1 (ARP-T1). These mutations were from the transcribed regions of encoding eRNAs [34, 35, 58] and were found to decrease ACTRT1 expression and enhancer activity. GLI1 promoter was found to immediately combine with ARP-T1, which inhibited GLI1 expression. Inhibition of ARP-T1 resulted in activation of the Hedgehog pathway in patients with Bazex Dupré Christol syndrome (BDCS). Moreover, they generated keratinocytes with insertions and deletions in the A2, B2, or CNE12 enhancer regions and found that disruption of these enhancers resulted in a decrease in ACTRT1 expression relative to cells containing the native (nonmutated) enhancer sequence. They also observed that enhancer mutagenesis increased the rate of keratinocyte proliferation. Taken together, these results suggest that BDCS is directly associated with loss-of-function mutations either altering the coding region of ACTRT1 or a disease mechanism in BCC involving mutations in eRNAs elements.

## 9. Perspectives and Future Directions

eRNAs emerged rapidly as an critical regulators of genetic transcription that interacted with the association of the enhancer and their targeted promoter sequences. Functional alterations of eRNAs have been reported to exert their effects on tumorigenesis in human tumors including breast cancer, CRPC, CRC, and basal cell carcinoma. Although eRNAs have been the focus of several researches, the functional role of eRNAs in transcriptional regulation and tumorigenesis has remained largely unknown. As the eRNAs field continues to explore, further research needs to be done to gain insight into the detailed mechanism of eRNAs in affecting enhancer activity/gene expression/tumorigenesis and the trending techniques to effectively investigate the relationship between aberrant eRNAs and tumorigenesis.

## Figures and Tables

**Table 1:** Enhancer RNAs in cancer and their fundamental characteristics.

Study	Cancer Type	Enhancer RNAs	Sample	Binding factors	Transcriptional orientation	Epigenetic characteristics
Li et al. (2013)	BC	TFF1, FOXC1, CA12, PGR SIAH2, KCNK5, P2RY2 SMAD7, GREB1, NRIP1	MCF-7	RNAPII, ER*α*	1D and 2D	High H3K4me1, low H3K4me3 and high H3K27Ac
Leveille et al. (2015)	BC	P21, TOB1, PRKAG2, SUFU	MCF-7	RNAPII, p300, p53	1D and 2D	High H3K4me1, low H3K4me3
Zhao et al. (2016)	CRPC	ARHGEF26, KLK15, HTR3A TLE1, SLC16A7	LNCaP, C4-2	RNAPII, p300, AR	2D	High H3K4me1, low H3K4me3
Zhao et al. (2016)	CRPC	FTO, LUZP2, MARC1 NCAM2	LNCaP, C4-2	RNAPII, p300, AR	2D	High H3K4me1, low H3K4me3
McCleland et al. (2016)	CRC	CCAT1	HT-29	RNAPII	2D	High H3K4me1, low H3K4me3
Bal et al. (2017)	BCC	ACTRT1	Keratinocyte	RNAPII	1D and 2D	High H3K4me1, low H3K4me3 and high H3K27Ac

BC: breast cancer; CRPC: castration-resistant prostate cancer; CRC: colorectal cancer; BCC: basal cell carcinoma.
